# Tunable piezoelectric PLLA nanofiber membranes for enhanced mandibular repair with optimal self-powering stimulation

**DOI:** 10.1093/rb/rbae150

**Published:** 2024-12-26

**Authors:** Shuo Chen, Xinqing Wang, Dong Zhang, Zhenhua Huang, Yina Xie, Fangping Chen, Changsheng Liu

**Affiliations:** Engineering Research Center for Biomedical Materials of Ministry of Education, East China University of Science and Technology, Shanghai 200237, P. R. China; Engineering Research Center for Biomedical Materials of Ministry of Education, East China University of Science and Technology, Shanghai 200237, P. R. China; Engineering Research Center for Biomedical Materials of Ministry of Education, East China University of Science and Technology, Shanghai 200237, P. R. China; Engineering Research Center for Biomedical Materials of Ministry of Education, East China University of Science and Technology, Shanghai 200237, P. R. China; Engineering Research Center for Biomedical Materials of Ministry of Education, East China University of Science and Technology, Shanghai 200237, P. R. China; Engineering Research Center for Biomedical Materials of Ministry of Education, East China University of Science and Technology, Shanghai 200237, P. R. China; Key Laboratory for Ultrafine Materials of Ministry of Education, School of Materials Science and Engineering, East China University of Science and Technology, Shanghai 200237, P. R. China; Engineering Research Center for Biomedical Materials of Ministry of Education, East China University of Science and Technology, Shanghai 200237, P. R. China; Key Laboratory for Ultrafine Materials of Ministry of Education, School of Materials Science and Engineering, East China University of Science and Technology, Shanghai 200237, P. R. China

**Keywords:** poly (l-lactic acid), piezoelectricity, electrospinning, self-powered, bone tissue repair

## Abstract

Poly (l-lactic acid) (PLLA) is a biocompatible, biodegradable material with piezoelectric properties, making it a promising candidate for providing self-powered stimulation to accelerate tissue repair. Repairs to various tissues, such as bone, cartilage and nerve, necessitate distinct piezoelectric characteristics even from the same material. However, the extensive utilization of PLLA piezoelectric scaffolds in various tissue is hindered by their low and single piezoelectric constants. In this study, PLLA nanofiber membranes with enhanced and adjustable piezoelectric constants (*d*_33_) were fabricated through oriented electrospinning. By carefully controlling the parameters of the spinning solution, a steady increase in *d*_33_ values from 0 to 30 pC/N was achieved. This advancement allows tailoring of PLLA nanofiber membranes to meet various piezoelectric requirements of different tissues. As an example of tailoring the optimal piezoelectric constants, we developed PLLA-2-0, PLLA-2-10, PLLA-2-15 and PLLA-2-20 nanofiber membranes with *d*_33_ values of 0, 5, 10 and 15 pC/N, respectively. The impact of varying piezoelectric constants of PLLA nanofiber membranes on cellular behavior and repair efficacy were validated through *in vitro* cellular experiments and *in vivo* mandibular critical defect repair. The results indicated that PLLA-2-20 demonstrated superior cell proliferation rate up to 130% and an osteogenic differentiation level approximately twice of the control. In addition, PLLA-2-20 significantly promoted cell adhesion and migration, and the cell aspect ratio was about five times higher than that of the control group. *In vivo*, PLLA-2-20 optimal restorative effects on rat mandibles via endogenous mechanical force-mediated piezoelectric stimulation, leading to complete histological restoration within 8 weeks. These findings highlight the potential of the PLLA membranes with high and adjustable *d*_33_ by a straightforward process. This study provides a novel approach for the development of highly electroactive membranes tailored to specific tissue repair needs.

## Graphical abstract

**Figure:**
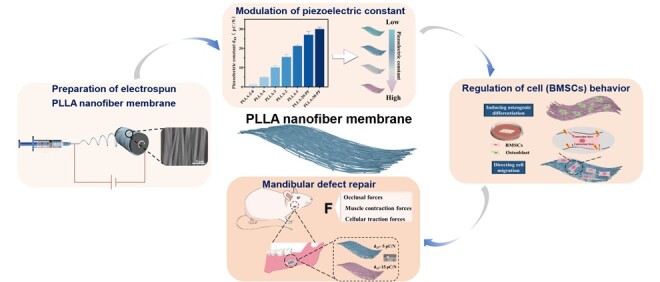

## Introduction

Piezoelectric materials possess the intrinsic capability to generate electrical charges or potentials in response to physiological loading [1, 2], cellular traction forces [3] or external ultrasound stimuli [4, 5]. These unique self-powered characteristics have demonstrated significant potential in tissue repair [6, 7]. It is noteworthy that there were differences in cellular behavior in response to the three stimulations. While ultrasound stimulation led to cell over-differentiation and reduced proliferation. Physiological mechanical forces, such as occlusal pressure in areas like the mandible, and cell traction in response to piezoelectric stimulation can stimulate cell proliferation and differentiation, respectively [8]. Therefore, the strategic application of loads can enhance the modulatory effects of piezoelectric materials on cells. 

Clinical studies suggest that electrical stimulation can shorten the healing of bone, nerves, blood vessels and other tissues, thereby promoting high-quality repair [9]. However, different regenerative situations require different intensities of electrical stimulation, necessitating the materials with tailored electrical properties for tissue repair. For instance, nerve repair benefits from targeted electrical stimulation with an optimal voltage of 100 mV/mm [10]. Vascular regeneration is more responsive to an optimal alternating voltage of 150 mV/mm [11, 12]. Bone tissue exhibits piezoelectric characteristics due to its collagen fibers [13–16]. Piezoelectric constants for the human femur is about 0.7 pC/N [17], and the tibia ranges from 7.8 to 8.7 pC/N [18]. According to Wolf's Law, the piezoelectric response in bone alters the electrical microenvironment and affects bone remodeling [19]. On high piezoelectric constants (10 pC/N), stem cells tend to differentiate into osteoblasts, whereas they differentiated into chondrocytes under low piezoelectric stimulation (3 pC/N) [20]. This highlights the significance of selecting the appropriate piezoelectric materials for bone repair, with a focus on having adjustable piezoelectric properties [20–22].

PLLA has garnered attention for its biocompatibility, biodegradability and piezoelectric properties [22, 23], making it a common choice for the fabricating piezoelectric periosteum to electrically stimulate bone regeneration. Nevertheless, PLLA’s low piezoelectric properties (about 9.8 pC/N [24]) limit its effectiveness in sensing cellular traction or minor physiological forces. To address this limitation, various strategies have been employed, including the incorporation of electrically active nanoparticles [25–28], polarization and stretching treatments [29]. The piezoelectric constant of PLLA was increased to 14 pm/V by doping graphene oxide (rGO) [30]. The piezoelectric constant of PLLA fibers increased significantly to 25 pC/N by stretching under an electric field, which is the highest value reported for PLLA [31]. However, these processes are highly intricate. It was urgent to develop simple and stable processes for preparing PLLA with adjustable piezoelectric properties.

Electrospinning is a widely esteemed technique for producing polymer fiber membranes [31–33], highly favored for its simplicity, cost-effectiveness and scalability [34]. The piezoelectric properties of polymers can be significantly enhanced through self-polarization and stretching effects during electrospinning. Studies have demonstrated that the enhancement of piezoelectric constants in PLLA is linked to the alignment of dipoles (C = O), which promotes the formation of an electroactive β-phase [20, 21, 35, 36]. Meanwhile, the properties of fibers are predominantly influenced by factors such as the spinning solution’s composition, the electric field’s intensity, the velocities of propulsion and acceptance, as well as various environmental conditions [37, 38]. Curry *et al.* [31] found that the piezoelectric properties of PLLA was in relation to acceptance speed. The *d*_33_ of PLLA exhibits an exponential increase as the diameter of fibers decreases [21]. It was shown that the piezoelectric properties of the PLLA can be adjusted by the electrospinning parameters. However, studies on PLLA membranes have primarily focused on maximizing the piezoelectric properties, with less exploration for the controlled preparation of piezoelectric constants [25, 39, 40]. The impact of materials with diverse piezoelectric properties on cell behavior and bone regeneration has not been thoroughly investigated.

**Figure 1.**
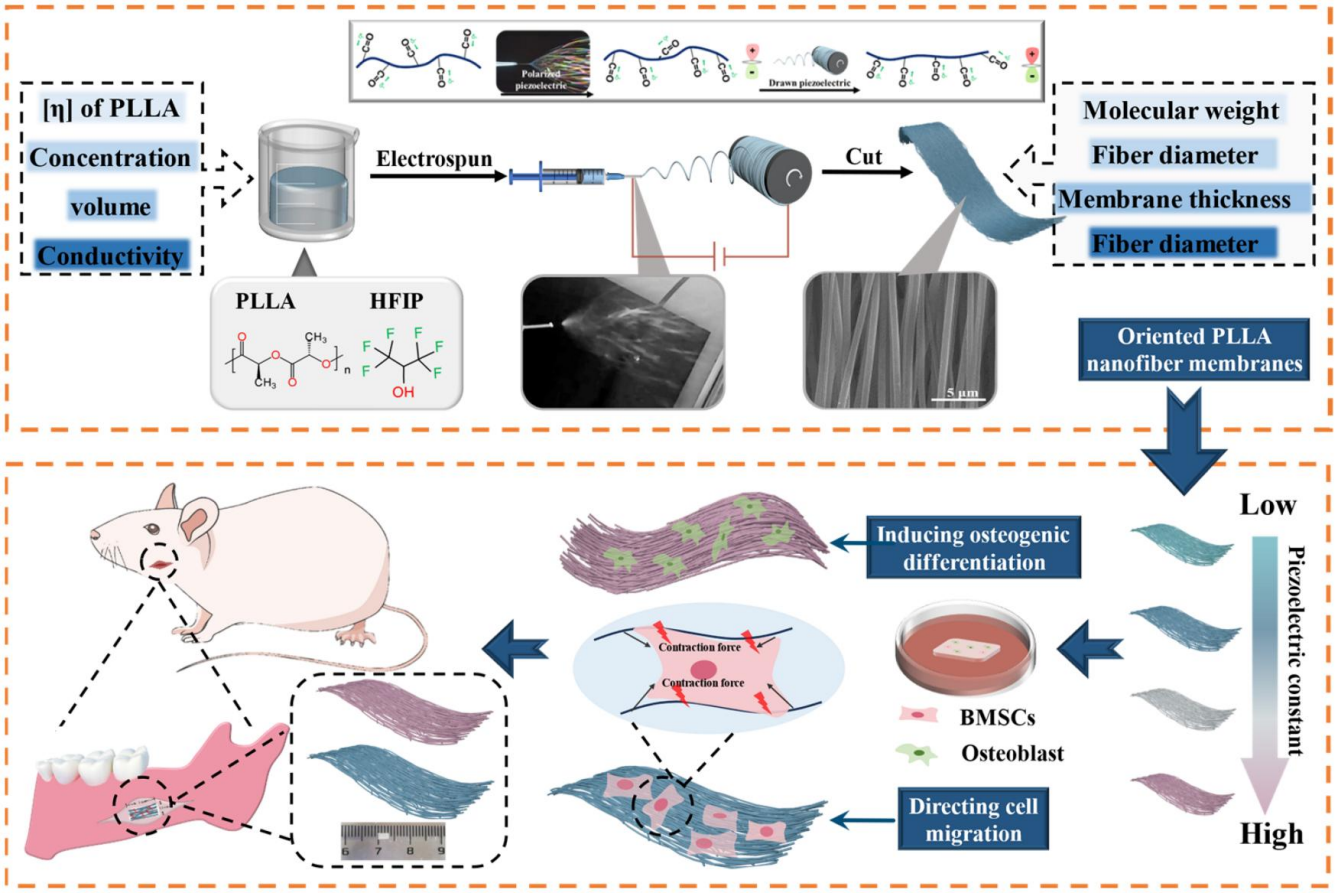
The fabrication process of degradable PLLA nanofiber membranes with adjustable piezoelectric constants and their induced differences in cell behavior and osteogenic activity.

Herein, oriented PLLA nanofiber membranes were successfully fabricated by electrospinning technology (Figure 1). The piezoelectric constants of these membranes were adjusted by altering the parameters of the PLLA spinning solution, such as the molecular weight of PLLA, the concentration, volume and conductivity of the spinning solution. In addition, the fiber structure, piezoelectric properties, crystalline phase structure and crystallinity of the PLLA nanofiber membranes were characterized. The results showed that the PLLA nanofiber membranes exhibit an oriented nanofiber morphology with adjustable piezoelectric constants ranging from 0 to 30 pC/N. *In vitro* cellular experiments were conducted to evaluate the different cellular responses of BMSCs on PLLA nanofiber membranes with various piezoelectric constants, including cell proliferation, migration and osteogenic differentiation. Subsequently, a rat mandibular defect model was established to assess the *in vivo* osteogenic effects of PLLA nanofiber membranes with different piezoelectric constants under endogenous load-mediated electromechanical stimulation. These findings offer insights for precise modulation of the piezoelectric constants in PLLA nanofiber membranes and their influence on specific cellular behaviors. This presents a novel approach for the development of highly electroactive periosteum.

## Materials and methods

### Fabrication of electrospun PLLA nanofiber membranes with controllable piezoelectric constants

#### Preparation of PLLA nanofiber membranes

A series of oriented PLLA electrospun nanofiber membranes were prepared by electrostatic spinning equipment (SS-X3, Beijing Ucalery Technology and Development Co., Ltd, China) and a high-speed receiver (3000 rpm). The spinning solution was regulated by the PLLA molecular weight, the concentration, conductivity and volume of PLLA spinning solution. Each of these solutions was injected into a 60-ml syringe fitted with a 27-gauge needle. A voltage of 12 kV was applied between the needle and the collector at 40°C and 20% relative humidity. The feed rate of the solution was controlled at 1 ml·h^−1^, with the distance between the needle and the receiver set at 15 cm.

#### Regulation spinning solution of PLLA nanofiber membranes

1. Molecular weight of PLLA. PLLA powders (Jinan Daigang Biotechnology Co., Ltd, China) with molecular weights of 130 000; 280 000; 530 000; 870 000, respectively, were dissolved in hexafluoro isopropanol (HFIP) (Aladdin, China). PLLA spinning solution was obtained after stirring overnight, and nanofiber membranes with different molecular weights were prepared according to the above method, named 1-PLLA, 2-PLLA, 3-PLLA, 4-PLLA, respectively.
2. Concentration of PLLA spinning solution. PLLA with a molecular weight of 870 000 was dissolved in HFIP and the concentration of spinning solution was varied to 4 wt.%, 3 wt.%, 2 wt.% and 1 wt.%. Different concentrations of nanofiber membranes were prepared by electrostatic spinning according to the same method, named PLLA-4, PLLA-3, PLLA-2, PLLA-1, respectively.
3. Volumes of PLLA spinning solution. PLLA-2 spinning solution was prepared according to (2), and the volume of the spinning solution was modified to be 10, 15, 20, 25, 30, 35 and 40 ml, respectively. PLLA nanofiber membranes were prepared by electrostatic spinning and named PLLA-2-10, PLLA-2-15, PLLA-2-20, PLLA-2-25, PLLA-2-3, PLLA-2-35, PLLA-2-40, respectively.
4. Conductivity of PLLA spinning solution. Equimolar pyridine (Aladdin, China) and formic acid (Aladdin, China) were mixed to formulate a PF solution with highly polar. High conductivity spinning solutions were obtained by adding 3 wt.% PF solution to PLLA-2-20 and PLLA-2-30. PLLA nanofiber membranes were prepared by electrostatic spinning and named PLLA-20-PF and PLLA-30-PF, respectively.

### Characterization of PLLA nanofiber morphology

Scanning electron microscopy (SEM) (S-4800, Hitachi, Japan) was used to observe the morphology of all PLLA nanofibers. To improve the electrical conductivity of the samples for observation, a thin layer of gold was sputtered on the surface for 90 s. The diameters of PLLA nanofibers were counted by Image J.

### Evaluation of piezoelectric property of PLLA nanofiber membranes

#### Quasi-static d_33_ meter

The piezoelectric constants of all PLLA nanofiber membranes were determined using a quasi-static d_33_ meter (ZJ-3; CAS; China). For each membrane, 10 points were randomly chosen for measurement. Schematic of the nanofiber membranes d_33_-test was shown in Figure S1.

#### Electrometer

A piezoelectric nanogenerator was constructed by fixing a PLLA nanofiber membrane (2 × 2 cm^2^) between two copper electrodes and encapsulating it with polyimide (PEI) tape. Periodic mechanical pressure (5 N) was applied to the PLLA nanofiber membranes using a linear motor. The resultant output voltage signals were measured and recorded by an electrostatic meter (Keithley 6514, Agitek Testing Co., China).

#### Piezo-response force microscopy

Piezoelectric properties of single PLLA nanofibers were characterized using piezo-response force microscopy (PFM). Briefly, PLLA nanofiber membranes were immobilized on a conductive substrate. An atomic force microscopy (AFM) (MFP-3D, Asylum Research, CA) was utilized to locate individual PLLA nanofibers in contact mode. An alternating voltage was applied across the fiber and the substrate via a probe (Muliti75, Asylum Research, USA). The corresponding changes in amplitude and phase were subsequently recorded.

### Crystal phase structure of different PLLA nanofiber membranes

Fourier-transform infrared spectroscopy (FTIR) (Nicolet 6700, Thermo Scientific, MA) was employed to analyze the phase content of all PLLA nanofiber membranes in the absorbance mode within the wavenumber range from 650 to 4000 cm^−1^. The crystalline phase of each sample was characterized using an X-ray polycrystalline diffractometer (XRD) (D8 Advance, AXS, Germany) over a 2θ range from 10° to 40°.

The phase transition temperatures of PLLA nanofiber membranes were determined using a differential scanning calorimeter (DSC) (DIAMOND, Platinum Elmer, China). The DSC curves were acquired by heating the samples (10 mg) from 20°C to 200°C at a heating rate of 10°C/min. The crystallinity index of each sample, corresponding to the acquired DSC curve, was calculated using the following equation:


$$X_{C}\;=\;\frac{\Delta H_{m\;}^{f}-\Delta H_{m}^{C}}{\Delta H_{100\%}^{f}}\times 100\%$$

where *X*c is the degree of crystallinity, Δ$H_{m}^{f}$ is the heat of melting (J/g) determined from the melting peak integral, Δ$H_{m}^{c}$ is the heat of cold crystallization (J/g) determined from the cold crystallization peak integral, and Δ$H_{100\%}^{f}$ is the heat of melting of 100% crystalline PLLA material (93.1 J/g [41]).

### Stem cell proliferation on PLLA nanofiber membranes

BMSCs were extracted from the femoral bone marrow of SD rats (Oricell^®^ Biotechnology Co., Ltd, China). Cells from generations 3–6 were used for *in vitro* cellular experiments. The BMSCs were maintained in growth medium (consisting of α-MEM solution supplemented with 10% FBS) and cultured at 37°C under 5% CO_2_. The PLLA nanofiber membranes were sterilized using Go-60 irradiation before cell inoculation. BMSCs were seeded at a density of 2000 cells per well on piezoelectric membranes, while tissue culture polystyrene plates (TCPs) served as controls to assess cell proliferation.

After incubation for the indicated durations (1, 3 and 5 days), CCK-8 (Beyotime Biomaterial Co., Ltd, China) reagent was added and the cells were further incubated at 37°C for 1 h. Optical density (OD) values were detected spectrophotometrically from the solution in the wells and the cell proliferation rate was calculated. Meanwhile, the live/dead state of the cells was observed using fluorescent staining, and images were captured with confocal laser scanning microscopy (CSM) (TCS-SP8, Leica, Germany).

### Morphology of BMSCs cultured on PLLA nanofiber membranes

Cells were seeded onto PLLA piezoelectric nanofiber membranes. After 24 h of culture, the cells were fixed with 4% paraformaldehyde (Tong-guang, China), and the cytoskeleton and nuclei of the attached cells were stained with phalloidin (FITC) (Thermo Scientific, MA) and 4,6-diamidino-2-phenylindole (DAPI) (Sigma, Germany), respectively. Additionally, adhesion proteins (Anti-Vinculin) (Abcam, China) on the cell surface were labeled with a specific anti-Vinculin antibody staining solution (Alexa Fluor^®^647) (Abcam, China). The adherence and morphology of the cells on the material were examined and documented using CLSM. The acquired images were then quantified with Image J.

### Stem cell differentiation on PLLA nanofiber membranes

BMSCs (5 × 10^4^ cells/well) were seeded onto PLLA piezoelectric nanofiber membranes in 24-well culture plates. The complete growth medium was replaced with osteo-inductive medium (Oricell^®^ Biotechnology, China) after 2 days. On Day 7 of osteogenic induction, the cells were assessed for the early osteogenic marker ALP using the ALP assay kit (Biotian Biomaterials Co., Ltd, China). Late-stage osteogenic mineralization was evaluated using Alizarin Red Staining (ARS). To facilitate staining, cells were incubated with a 10% cetylpyridine (Aladdin, China) solution for 2 h. OD values were measured to quantify the presence of calcium nodules, indicating osteogenic activity. Images of the stained cells were captured using a digital camera.

For gene expression analysis, RT-qPCR was employed to assess the osteogenic differentiation of BMSCs on PLLA nanofiber membranes with different piezoelectric constants. BMSCs (1 × 10^5^ cells/well) were cultured on PLLA piezoelectric membranes in 6-well plates, and an osteogenic induction medium was introduced on Day 2. After 7 days, the expression levels of ALP, bone morphogenetic protein 2 (BMP-2), collagen 1 (Col-1), osteocalcin (OCN), osteoprotegerin (OPN), runt-related transcription factor 2 (Runx-2) and transforming growth factor (TGF-β) genes were detected. GAPDH was utilized as an internal reference gene (Table S1).

### Evaluation of the osteogenic activity of PLLA nanofiber membranes by implantation

The mandibular critical defect model was used to assess the effect of varying PLLA piezoelectric constants on bone regeneration. This animal experimentation protocol was approved by the Animal Ethics Committee of the East China University of Science and Technology (ECUST-21046). Eight-week-old SD rats were selected to create a bilateral mandibular defect model.

The PLLA-2-10 and PLLA-2-20 were sterilized via Co-60 irradiation, the untreated defect group was used as a control (CON). The rats were evenly divided into three groups (*n* = 6 per group) based on the implanted membranes: (i) no treatment group (CON); (ii) PLLA-2-10 group; and (iii) PLLA-2-20 group. Bone mineral density (BMD), bone mineral content (BMC), bone volume fraction (BV/TV) and trabecular separation (Tb. Sp) were assessed at the defect site using micro-computerized tomography (Micro-CT) scanning (Skyscan 1072, Belgium). Subsequently, the mandibular samples were decalcified and sections were prepared in the sagittal plane of the mandibular defect for hematoxylin and eosin stain (H&E) and Masson’s trichrome stain (MTS).

### Statistical analysis

Experimental data were expressed as mean ± standard deviation. One-way ANOVA analysis was conducted to evaluate the significance of mean differences among groups. Additionally, two-way AVONA analysis was utilized to evaluate the interaction effects between two independent variables on the dependent variable. The value bars with ns were not considered significant, asterisk denoted statistically significant differences * *P* < 0.05, **P < 0.01, ***P < 0.001 and ****P < 0.0001.

## Results and discussion

### PLLA nanofiber membranes with enhanced and different piezoelectric constants

Theoretically, the piezoelectricity of PLLA is attributed to the ordered arrangement of C=O dipoles on its molecular chain, and this ordered molecular chain configuration contributes to the formation of the electrically active β crystalline phase [21]. In this study, piezoelectric constants of PLLA were regulated by the molecular weight of PLLA, the diameter of fiber, the thickness of nanofiber membranes, and the conductivity of the electrospinning solution.

#### Piezoelectric constants of PLLA nanofiber membranes modulated by molecular weight of PLLA

Modulating the density of C=O dipoles along the polymer backbone, a parameter that can be achieved by changing the molecular weight of PLLA, potentially improves the piezoelectric properties. 1-PLLA, 2-PLLA, 3-PLLA and 4-PLLA, with uniform fiber diameters of about 600 nm, were successfully prepared (Figure S2A). The piezoelectric constants of these PLLA nanofiber membranes were measured on Day 0 and 7 (Figure S2B). It was found that the piezoelectric constants of these nanofiber membranes increased with the molecular weight of PLLA. In particular, at Day 0, 4-PLLA with a high molecular weight of 870 000 exhibited a piezoelectric constant about twice that of 1-PLLA with a low molecular weight of 130 000. Even after being exposed to air for 7 days, the *d*_33_ value of 4-PLLA remained 10.9 pC/N, approximately 1.3 times higher than that of 1-PLLA. Based on these findings, 4-PLLA was chosen for further experiment, with the aim of achieving a higher piezoelectric constant of the membrane.

#### Piezoelectric constants of PLLA nanofiber membranes modulated by the concentration of the spinning solution

**Figure 2.**
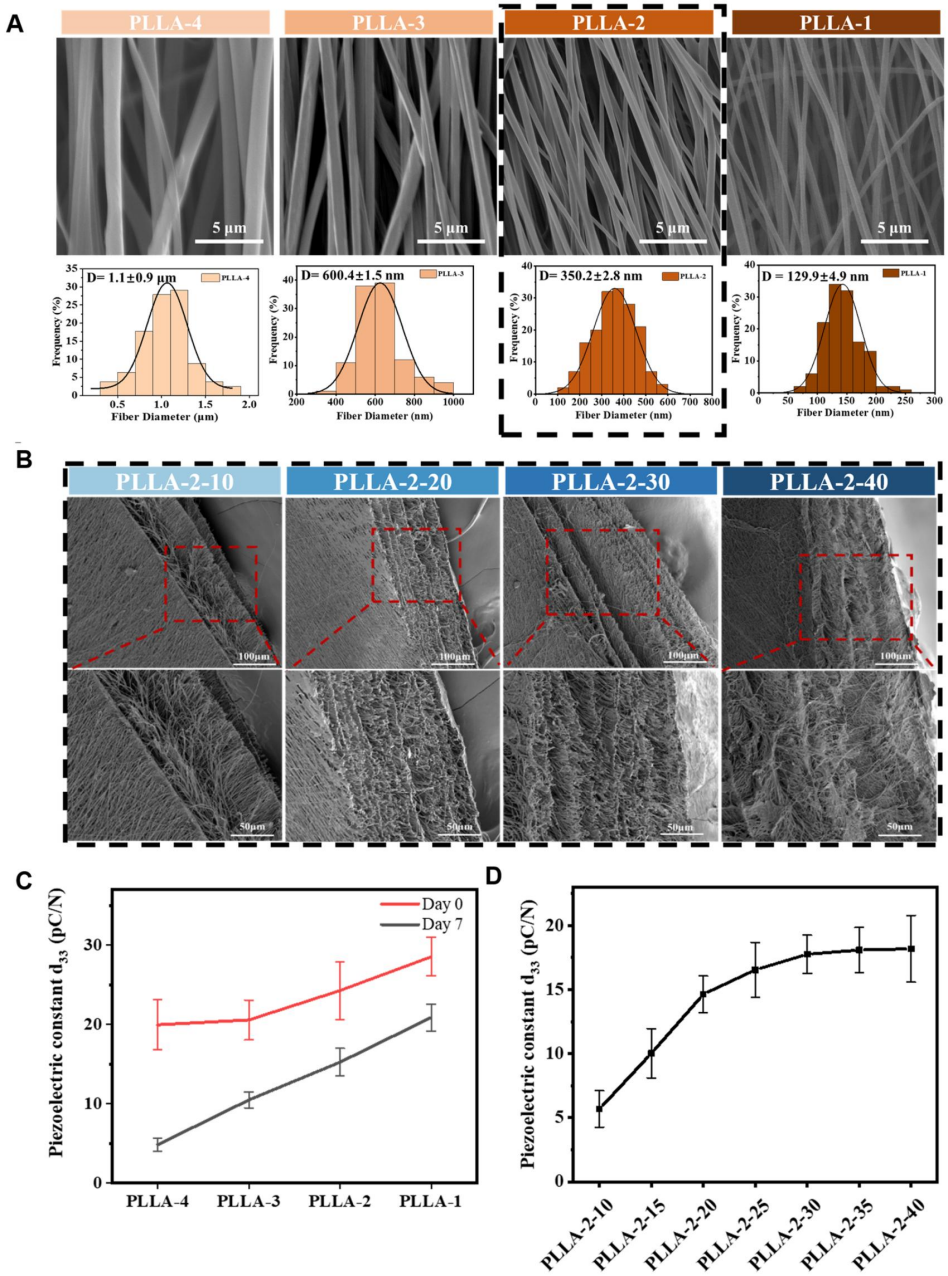
Microscopic morphology and piezoelectric properties of PLLA nanofiber membranes. (**A**) Morphological observation and diameter distribution of PLLA nanofibers by modulating the concentration of PLLA spinning solution. (**B**) Morphological observation of PLLA nanofiber membranes by modulating the volume of the spinning solution with the same concentration. (**C**) Piezoelectric constants of PLLA nanofiber membranes controlled by spinning concentration. (**D**) Piezoelectric constants of PLLA nanofiber membranes controlled by modulating the volume of the spinning solution (mean ± SD, *n* = 10).

By varying the mass concentration (4, 3, 2, 1 wt.%) of PLLA spinning solution, PLLA-4, PLLA-3, PLLA-2 and PLLA-1 were prepared respectively. Figure 2A shows that the fiber diameters of these membranes were 1100, 600, 350 and 130 nm, respectively. In addition, PLLA nanofiber membranes with different concentration showed predominantly oriented fiber morphologies. Notably, the fiber distribution in PLLA-1 was more random, which may be attributed to the influence of airflow generated by the high-speed rollers during the spinning process. Such airflow can disrupt the fiber arrangement, resulting in microscopic misalignment and macroscopic fluttering of the fibers. For the stability of the electrospinning process, the PLLA-2 sample was selected to initiate the experiment (indicated by the dashed line in Figure 2A) to further enhance the *d*_33_ of the PLLA nanofiber membrane.

On Day 0, the piezoelectric constants (*d*_33_) of PLLA-1; PLLA-2; PLLA-3; PLLA-4 nanofiber membranes with different concentrations (1, 2, 3 and 4 wt.%) were measured to be 28.5, 24.3, 19.9 and 19.7 pC/N, respectively (Figure 2C). On Day 7, the *d*_33_ values of these membranes were stable at 21.4, 15.5, 10.7 and 5.1 pC/N, respectively. Figure S3 shows that the piezoelectric constants of these membranes were monitored continuously over the subsequent 4 weeks. It can be seen that the reduction of spinning solution concentration can significantly enhance the piezoelectric constants of PLLA nanofiber membranes, and the *d*_33_ of PLLA-4, PLLA-3, PLLA-2 and PLLA-1 were basically controlled at values of 5, 10, 15 and 20 pC/N.

#### Piezoelectric constants of PLLA nanofiber membranes modulated by volume of spinning solution

Based on the above results, PLLA-2 nanofiber membranes with stable spinning parameters and high piezoelectric constants of 15.5 pC/N were selected to investigate the effect of membrane thickness on piezoelectric properties. Here, the thickness of the nanofiber membrane was regulated by changing the volume of the PLLA-2 spinning solution. Figure 2B displayed the cross-sectional morphology of PLLA-2-10, PLLA-2-20, PLLA-2-30, PLLA-2-40. Table S2 showed that the thicknesses of these membranes were approximately 66.6, 190.1, 276.6 and 385.1 μm, respectively. As shown in Figure 2D, the *d*_33_ of PLLA nanofiber membranes increases linearly with thickness. For example, the *d*_33_ values were about 5.6 pC/N for PLLA-2-10, 10 pC/N for PLLA-2-15, and 14.7 pC/N for PLLA-2-20. However, as the volume of PLLA-2 spinning solution was further increased, the increase of *d*_33_ slowed down and reached a maximum value of 18.7 pC/N for PLLA-2-40. It was found that the piezoelectric constant of PLLA-2-40 was about 3.2 times that of PLLA-2-10.

#### Piezoelectric constants of PLLA nanofiber membranes modulated by the conductivity of the spinning solution

PLLA-2-20 and PLLA-20-PF were characterized to evaluate the effect of conductivity on fiber morphology and diameter. It can be seen in Figure S4A, the increase in conductivity brings about a decrease in fiber diameter, which is approximately 275 nm for PLLA-20-PF, a 24.7% decrease compared to PLLA-2-20. Even after being exposed to air for 7 days, the *d*_33_ of PLLA-20-PF was 26.8 pC/N (Figure S4B), which was 1.7 times higher than that of PLLA-2-20. At the same time, the piezoelectric constant of PLLA-30-PF, which prepared by adding PLLA-2-30–3 wt.% PF solution, as high as 30.7 pC/N. This enhancement is mainly attributed to two reasons: the fibers are finer and more polar, thus effectively maintained the effect of electrostatic spinning, and the increased sensitivity of fibers to external forces, resulting in a higher piezoelectric response. As a result, PLLA-20-PF responds with more piezoelectric charge and a higher piezoelectric constant under the vibration force of about 0.35 N of the measuring instrument.

In summary, the piezoelectric constant of PLLA nanofiber membranes were optimized by changing the properties of the PLLA spinning solution. Among them, the PLLA-30-PF piezoelectric constant was stable with 30.7 pC/N, which are close to the theoretical peak of the piezoelectric constant of the PLLA fiber model (45 pC/N [30]). And this value exceeds the highest recorded value of PLLA piezoelectric constant of 25 pC/N [30, 42]. Furthermore, the PLLA nanofiber membranes exhibited adjustability within the range of 0–30 pC/N. For instance, samples with piezoelectric constants around 5 pC/N are PLLA-4 and PLLA-2-10, while the piezoelectric constants of PLLA-3 and PLLA-2-15 membranes are 10 pC/N, and likewise, samples with piezoelectric constants of 15, 20, 25 and 30 pC/N have corresponding nanofiber membrane samples that can be repetitively prepared (Table S3).

**Figure 3.**
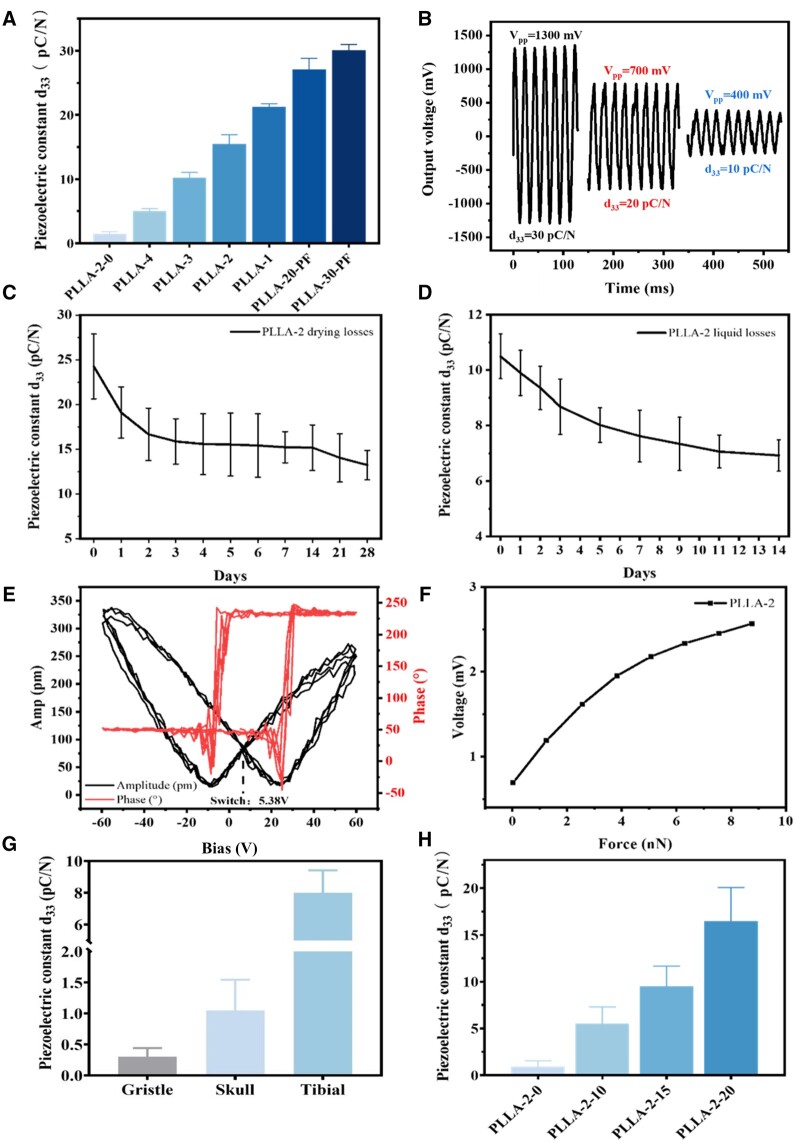
Piezoelectric properties of PLLA nanofiber membranes. (**A**) Plot of the range of piezoelectric constants and their corresponding samples. (**B**) The output voltage of a PLLA nanofiber membrane with three different *d*_33_ under the same treatment conditions. (**C**) Piezoelectric stability of PLLA nanofiber membranes in dry environments. (**D**) Piezoelectric stability of PLLA nanofiber membranes in liquid environments. (**E**) PFM amplitude and phase lag loop of PLLA-2 nanofiber membranes. (**F**) Output voltage profiles of a single PLLA-2 nanofiber subjected to a certain force. All measurements were recorded in residual mode to avoid electrostatic contributions. (**G**) Piezoelectric constants of human cartilage [43], skull [17] and tibia [18]. (**H**) Piezoelectric constants of PLLA nanofiber membranes used in the experiment and their corresponding sample names (mean ± SD, *n* = 10).

By varying the characteristics of PLLA electrospinning solution, such as PLLA molecular weight, the concentration, volume and conductivity of the spinning solution, the piezoelectric constants of the nanofiber membranes can be adjusted within the range of 0–30 pC/N, as detailed in Figure 3A and summarized in Table S3. Notably, the *d*_33_ of PLLA-2-0 is approximately 0.3 pC/N, which refers to PLLA-2-10 exposed to air for 12 months. It can be seen from Figure 3B, PLLA-3, PLLA-1 and PLLA-30-PF with *d*_33_ values about 10, 20 and 30 pC/N exhibited output voltages of 400, 700 and 1300 mV, respectively. The results showed that PLLA nanofiber membranes had stable alternating voltage outputs. Under the same force, the larger the piezoelectric constant, the higher the output voltage of the membrane.

As illustrated with PLLA-2 as a representative case, the piezoelectric stability of nanofiber membranes was evaluated through consecutive measurements of *d*_33_ both in dry conditions and in α-MEM medium. After 3 days, the *d*_33_ of PLLA-2 reached a stable value of 15.7 pC/N and maintained this value for about 4 weeks (Figure 3C). Even in a liquid environment, the *d*_33_ value remained at 66.7% of the initial value after 2 weeks, decreasing from 10.4 to 6.9 pC/N (Figure 3D), which is promising for the application of PLLA nanofiber membranes in liquid environments such as inside organisms.

The sensitivity of piezoelectric materials to electromechanical transformations is crucial for their widespread use. The piezoelectric properties of a single fiber were characterized by PFM. The amplitude and phase angle hysteresis loops under alternating voltage, as well as PFM images of morphology, amplitude and phase for PLLA-2 were shown in Figure 3E and Figure S5. The piezoelectric nature of PLLA nanofibers was evidenced by the characteristic butterfly amplitude and 180° phase transition. Meanwhile, the piezoelectric response switch for PLLA is located around +5 V, which is consistent with the literature findings [36]. Figure 3F shows the high sensitivity of the piezoelectric response of PLLA-2 nanofiber, when subjected to a force of 10 nN, produced an output voltage of about 2.5 mV. With this high piezoelectric sensitivity, the PLLA fiber membrane can sense the cellular traction force (about tens of nN [44]) and output an electrical signal. Therefore, the piezoelectric material can modulate cell behavior by electrical stimulation *in situ*, suggesting potential for implantation *in vivo* and participation in the regulation of electrical signals. The piezoelectric properties of the bones of the human body are different (Figure 3G), where the piezoelectric constant of the tibia is about 8 pC/N. Based on this, the piezoelectric constants of the PLLA nanofiber membranes were set to be 0, 5, 10 and 15 pC/N in this study. To ensure the uniform fiber diameters of PLLA piezoelectric membranes, PLLA-2 samples with different thicknesses were selected as follows (Figure 3H): PLLA-2-10 (*d*_33_ = 5.6 pC/N), PLLA-2-15 (*d*_33_ = 10 pC/N), and PLLA-2-20 (*d*_33_ = 14.7 pC/N), with PLLA-2-0 as a non-piezoelectric control. The effects of different piezoelectric constants for PLLA nanofiber membranes on cell behavior and osteogenic activity *in vitro* and *in vivo* were explored.

### Crystalline phase of PLLA nanofiber membranes with different piezoelectric constants

Differences in piezoelectric constants of PLLA nanofiber membranes stem from variations in the content of the electroactive β-phase. The crystallization and phase structures of PLLA nanofiber membranes with different piezoelectric constants were analyzed by IR, XRD, and DSC.

**Figure 4.**
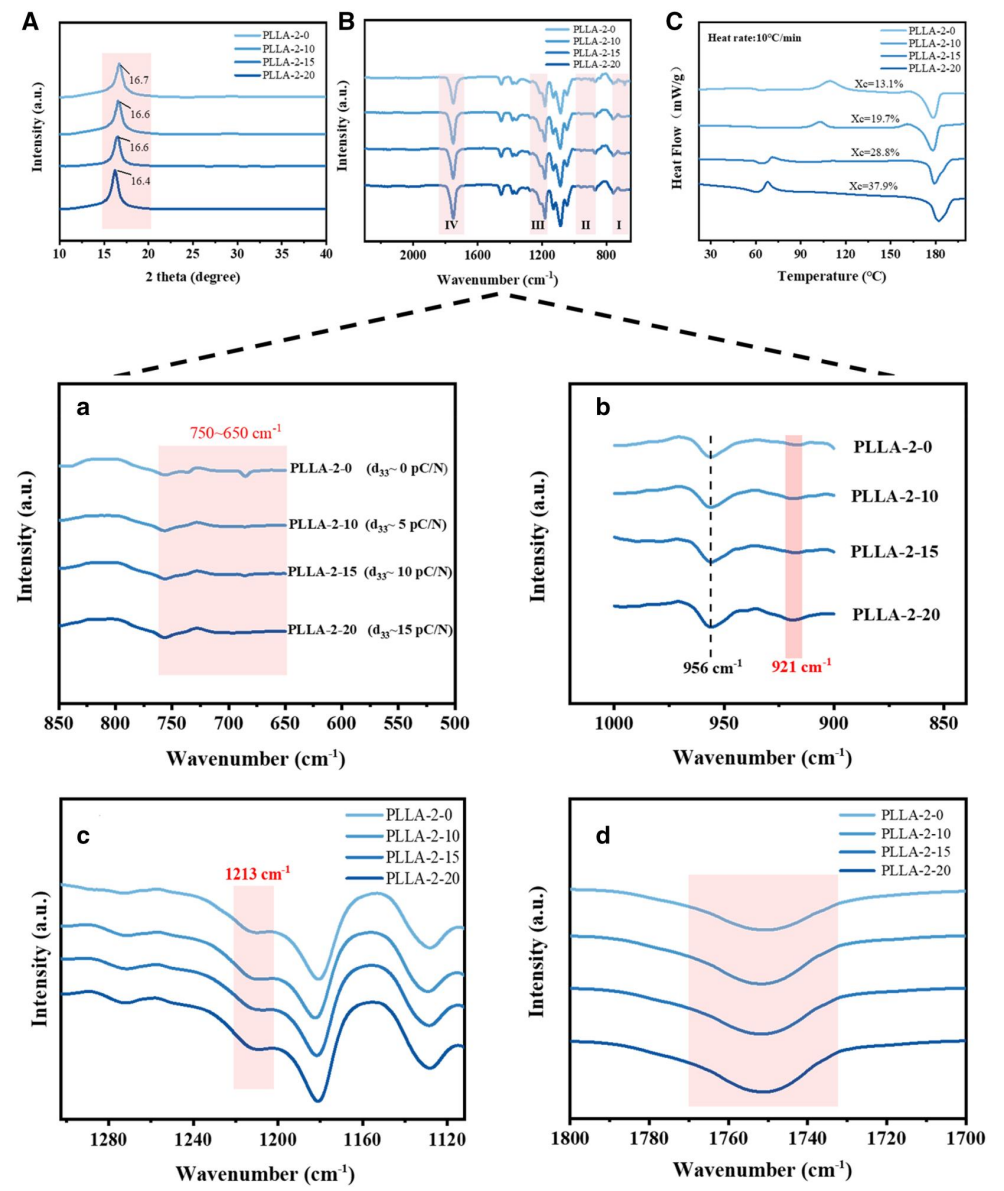
Structural analysis of PLLA nanofiber membranes with different piezoelectric constants. (**A**) XRD patterns of PLLA-2-0, PLLA-2-10, PLLA-2-15 and PLLA-2-20. (**B**) FTIR spectral images with highlighted regions: (a) 850–500 cm^−1^; (b) 1100–800 cm^−1^; (c) 1300–1100 cm^−1^; (d) 1800–1700 cm^−1^. (**C**) DSC curves for PLLA-2-0, PLLA-2-10, PLLA-2-15 and PLLA-2-20.

Figure 4A shows the XRD patterns of PLLA nanofiber membranes with sharp peaks indicating a crystalline phase for all samples. The PLLA-2-0 membrane’s diffraction peak at 16.7° (220/100) corresponded to the α-phase, while the strong signals for PLLA-2-10 and PLLA-2-15 at 16.6° (110) confirmed a predominantly β-phase [21, 36]. Notably, the PLLA-2-20 membrane with high piezoelectric constants of 15 pC/N had diffraction angles at 16.4° (131), suggesting an increased share of the polar phase.

FTIR analysis (Figure 4B) revealed that α-crystalline phase of PLA, known for its thermal stability, exhibited multiple energy band splitting in the 750–650 cm^−1^ region, whereas the polar β-crystalline phase had a simpler spectrum [36]. With the increase of *d*_33,_ the spectra in the region of 750–650 cm^−1^ band (Figure 4B) became less complex_,_ particularly for PLLA-2-20, which has predominantly β crystalline phase and smoother spectral bands. Additionally, PLLA-2-20 membranes with higher piezoelectric constants showed a decrease in the peak area at 956 cm^−1^, which is the amorphous phase, and the absorption band at 921 cm^−1^ was visible, characteristic of the β crystalline phase for PLLA (Figure 4B). In addition, the band at 1213 cm^−1^, indicative of the -CH_3_ and -C=O vibrations and the crystalline phase (α + β) absorption peak, is present in all samples and intensified in the PLLA-2-20 membrane (Figure 4C). Finally, the broad band near 1750 cm^−1^, attributed to the C=O stretching vibration [36], is consistent across all samples, confirming the presence of the β phase (Figure 4D).

DSC analysis (Figure 4C) indicated that an increase in crystallinity from 13.1% to 37.9% in PLLA nanofiber membranes contributed to an increase in the piezoelectric constant. It was in consistent with the results of FTIR. The results of the crystallographic phase analysis indicate that the enhancement in the piezoelectric constant of the PLLA nanofiber membrane is linked to the transition of the crystalline phase to the polar β-phase. Additionally, the rise in overall crystallinity leads to an increase in the content of the β-phase.

### Cell growth on different piezoelectric PLLA nanofiber membranes

**Figure 5.**
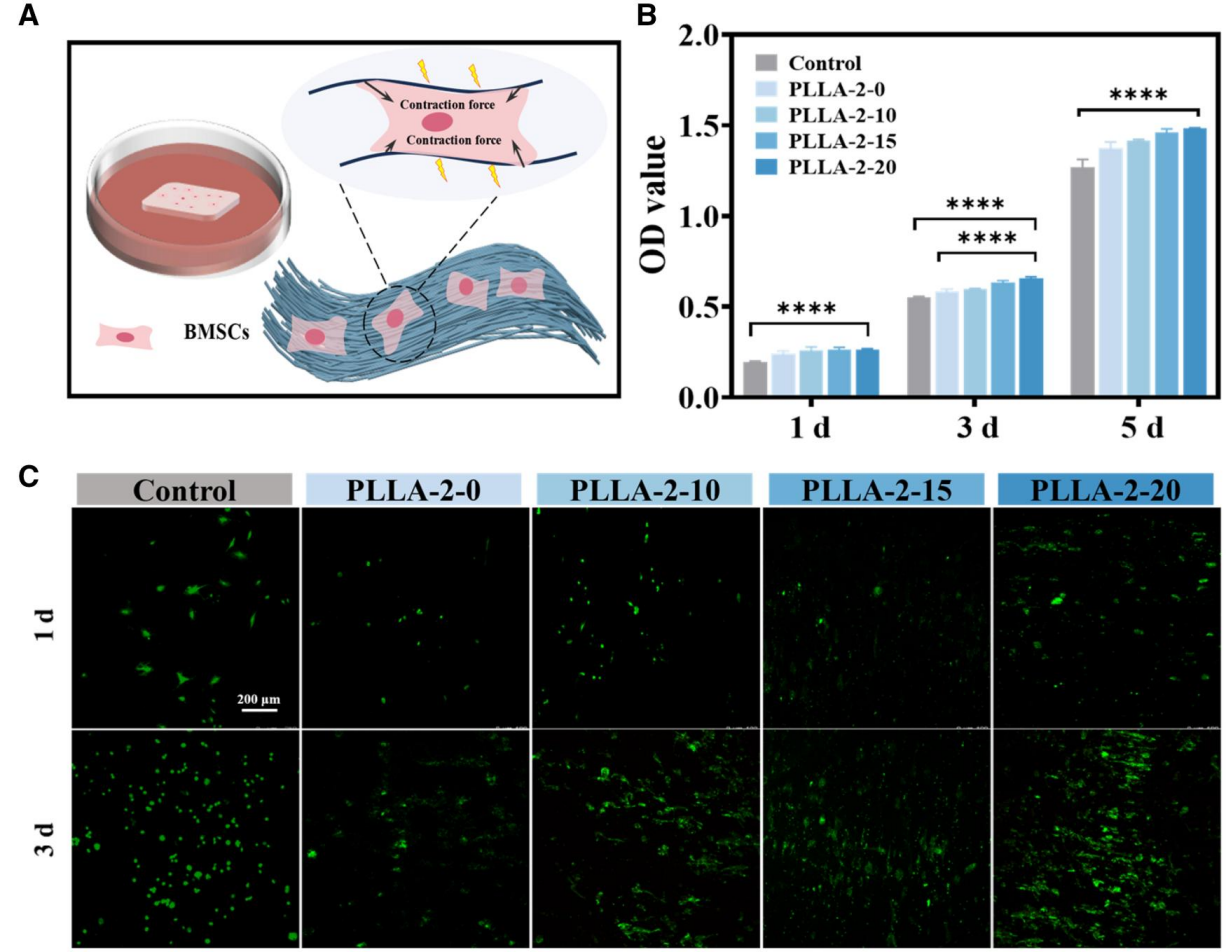
Cellular response of BMSCs on PLLA nanofiber membranes with different piezoelectric constants. (**A**) Schematic diagram of cell-material interactions. (**B**) OD values at 1, 3 and 5 days by CCK-8 method. (**C**) Fluorescence staining images of BMSCs cultured on membranes at 1 and 3 days (mean ± SD, *n* = 3, *P < 0.05, **P < 0.01, ***P < 0.001 and ****P < 0.0001).

When stem cells are cultured on piezoelectric nanofiber membranes, they initially establish contact with the nanofibers to form focal adhesions. These oriented nanofibers then guide cell migration and growth (Figure 5A). During the cell migration, they exerted a reaction force on the nanofibers due to intracellular tension. The piezoelectric nanofibers responded to the force by deforming and generating an electrical signal *in situ*. As PLLA-2-0, PLLA-2-10, PLLA-2-15 and PLLA-2-20 nanofiber membranes possessed varying piezoelectric constants (*d*_33_ approximately 0, 5, 10 and 15 pC/N), their response to applied forces and the resulting electrical signals also differ, leading to variations in cell behavior.

To assess the effect of different piezoelectric stimulations on cell proliferation, CCK-8 and live/dead fluorescence staining were used. The results showed that the OD value increased with increasing piezoelectric constant (Figure 5B). Further analysis of the cell proliferation rate (Figure S7) revealed that the cell proliferation rate increased with the increase of *d*_33_. Notably, in the PLLA-2-20 group with *d*_33_ of 14.7 pC/N, the proliferation rate exhibited a significant enhancement, reaching a value-added rate of up to 130%. The effect of PLLA nanofiber membranes with different piezoelectric constants on cell proliferation was graphically represented in Figure 5C. It is evident that the PLLA-2-20 group displayed a higher number of live cells spreading compared to PLLA-2-0. Based on the above results, it is clear that the positive correlation between the piezoelectric stimulation intensity and the promotion of BMSCs proliferation, with the PLLA-2-20 membranes demonstrating the most pronounced effect.

**Figure 6.**
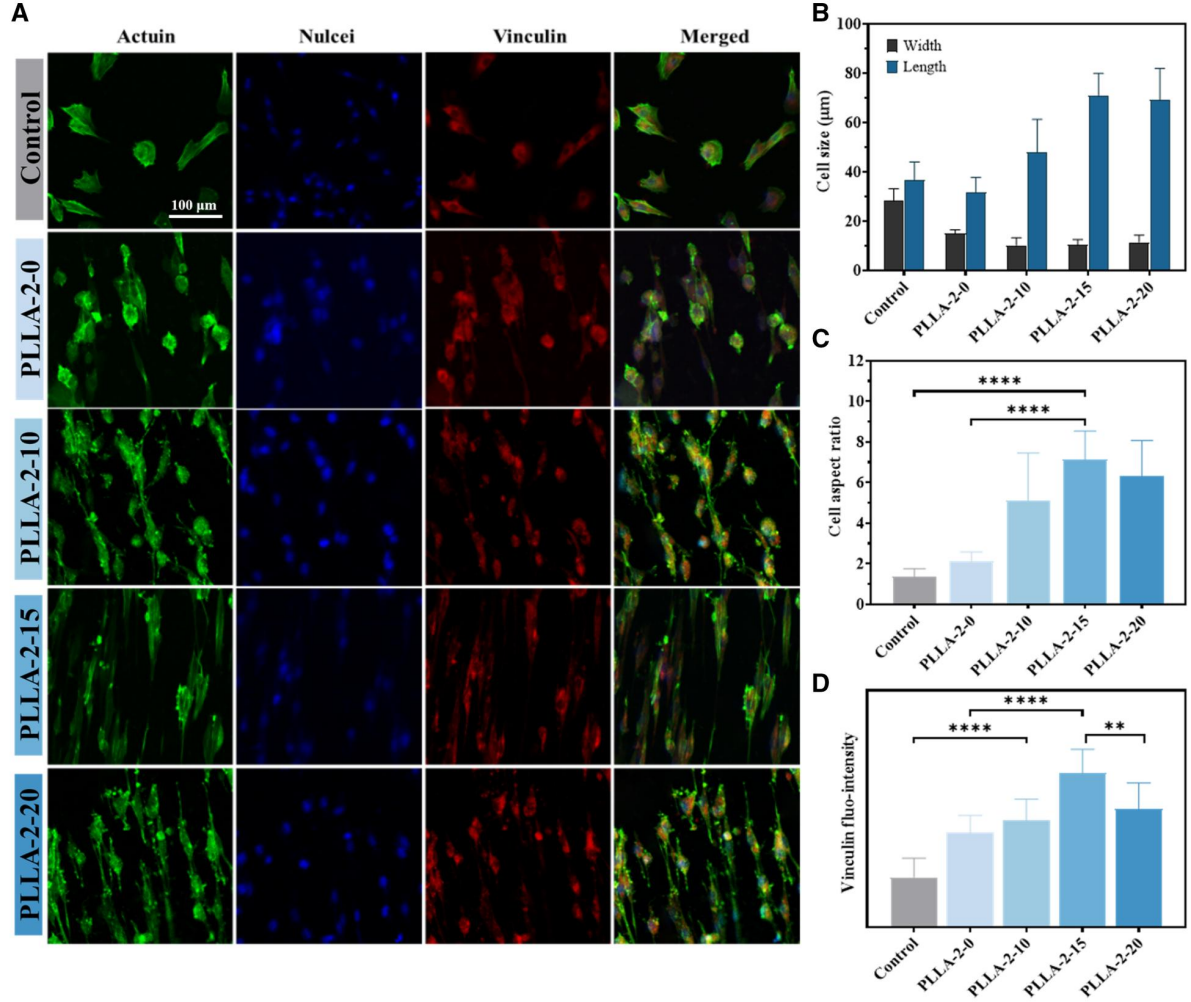
Adhesion and migration behavior of cells on membranes with different piezoelectric constants. (**A**) Immunofluorescence staining images of BMSCs cultured for 24 h. Quantitative analysis of (**B**) cell growth in length and width, (**C**) cell aspect ratio, (**D**) Vinculin fluorescence intensity. Asterisks indicate statistically significant differences (mean ± SD, *n* = 3, *P < 0.05; **P < 0.01; ***P < 0.001; ****P < 0.0001).

The oriented structure of PLLA nanofibers guides and promotes the directional migration of cells. Meanwhile, electrical stimulation from the piezoelectric PLLA nanofiber membranes modulates the local influx of Ca^2+^ within the cell membrane. This uneven charge distribution on the membrane surface triggers cell contraction and protrusion, ultimately inducing cell migration [8]. Therefore, the PLLA-2-0, PLLA-2-10, PLLA-2-15 and PLLA-2-20 nanofiber membranes with different piezoelectric constants can modulate cell morphology and influence cell migration. To assess cell adhesion spreading, the nuclei, cytoplasm and Vinculin proteins were stained (Figure 6A). Compared with free-growing cells in TCPs, cells cultured on oriented PLLA nanofiber membranes showed a pronounced stretching effect along the fiber orientation direction. Notably, the PLLA-2-15 membrane with *d*_33_ of 10 pC/N, was particularly effective in guiding the directional extension of cells, achieving a cell aspect ratio of 8:1 (Figure 6B and C), which was significantly higher than the aspect ratio of about 1.5:1 of the cells grown in standard culture plates. Interestingly, the capacity to modulate cell migration did not increase uniformly with the piezoelectric constant. The PLLA-2-20 membrane, despite having a higher *d*_33_ of 15 pC/N, was not as effective as the PLLA-2-15 membrane in inducing cell migration, with a cell aspect ratio decreased to 6:1. Vinculin protein staining and quantitative analysis showed that cells on PLLA-2-15 membranes displayed the highest fluorescence intensity, approximately three times higher than that of cells in the TCPs (Figure 6D).

Therefore, it can be concluded that the difference in piezoelectric constants of PLLA nanofiber membranes can modulate cell adhesion and migration, with a piezoelectric constant of about 10 pC/N (PLLA-2-15) being optimal for maximizing these effects, followed by the PLLA-2-20 membrane. In conclusion, electrical stimulation can enhance cell migration and proliferation, which is crucial for stem cell recruitment. This approach establishes a foundation for regulating cell differentiation.

### Cell differentiation on different piezoelectric PLLA nanofiber membranes

**Figure 7.**
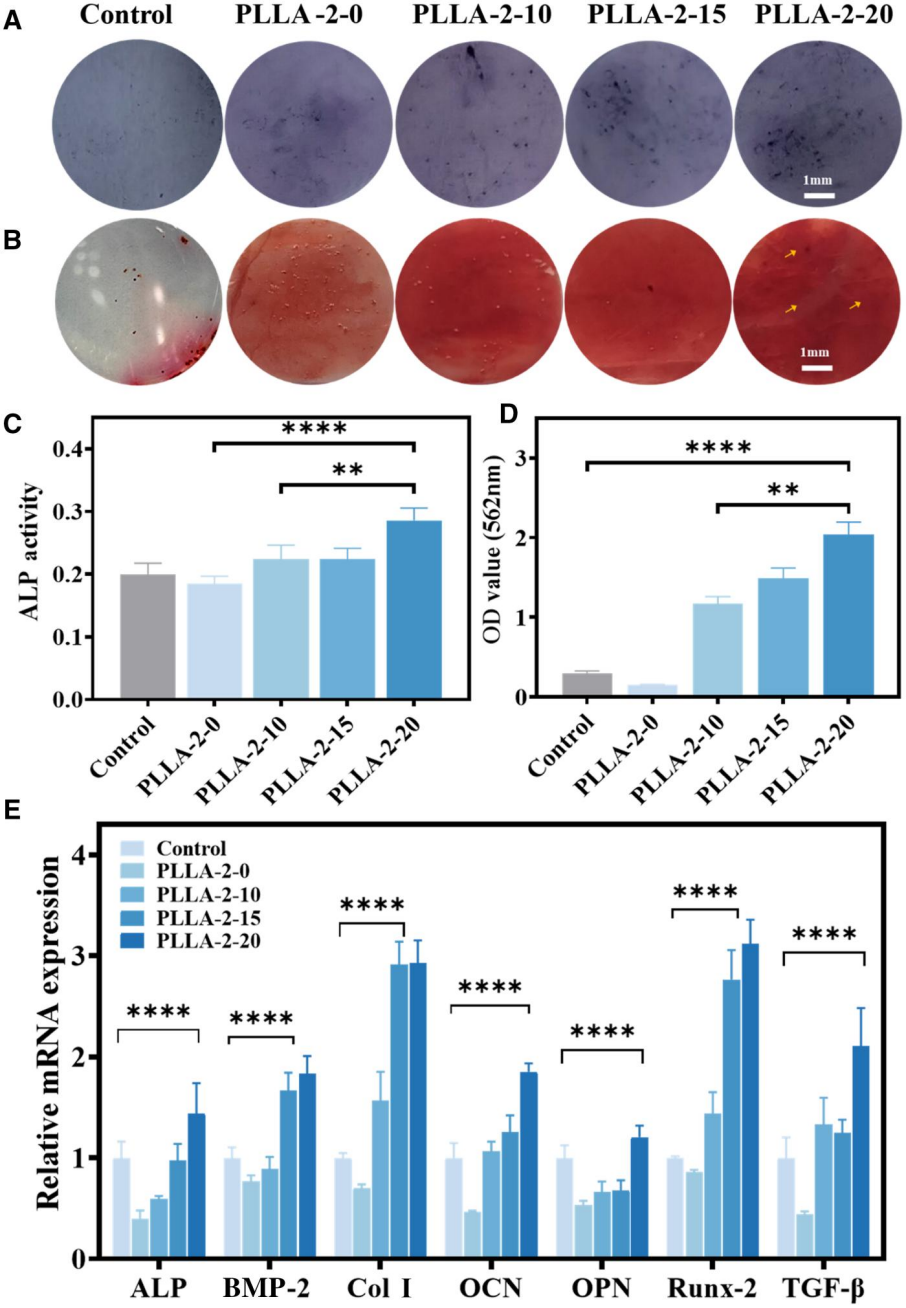
Osteogenic differentiation of BMSCs on PLLA membranes with different piezoelectric constants. (**A**) ALP staining images after 7 days incubation. (**B**) ARS staining after incubation for 14 days for mineralization assessment. (**C**) Relative activity of ALP. (**D**) Intensity of staining for calcium deposition. (**E**) RT-qPCR analysis of genes (ALP, BMP-2, Col I, OCN, OPN, Runx-2 and TGF-β) expression after incubation for 7 days (mean ± SD, *n* = 3, *P < 0.05, **P < 0.01, ***P < 0.001, ****P < 0.0001).

Differences in piezoelectric constants significantly influence osteogenic differentiation. ALP activity is a key early indicator of osteogenesis. ARS staining of calcium nodules indicates late-stage osteogenic activity. BMSCs were seeded onto PLLA-2-0, PLLA-2-10, PLLA-2-15 and PLLA-2-20 nanofiber membranes, and then induced in culture for 7 days. Figure 7A showed PLLA nanofiber membrane’s effect on early osteogenesis by ALP staining. An increase in the piezoelectric constant corresponded with an expansion of the ALP-positive area on the membrane. Quantitative analysis in Figure 7C showed that PLLA-2-20 with the highest piezoelectric constants of 15 pC/N had elevated ALP protein activity. Figure 7B showed calcium nodules stained with ARS. The mineralization rate was significantly higher in PLLA-2-20 characterized by a dense accumulation of dark-colored calcium deposits. Figure 7D showed that the OD values of the PLLA-2-20 were approximately 10-fold higher than the control and 2-fold than the PLLA-2-0 and PLLA-2-10. This indicated that the high-intensity piezoelectric response significantly induced late osteogenic differentiation of BMSCs.

Figure 7E shows the gene expression levels of osteogenic-specific matrix proteins were assessed by RT-qPCR. The PLLA-2-0 and PLLA-2-10 with the piezoelectric constant about 0 pC/N and 5.6 pC/N, exhibited lower expressing of osteogenic-related genes than that of the control. In line with ALP activity, PLLA-2-20 with a high piezoelectric constant of 14.7 pC/N exhibited the highest expression of early osteogenic ALP genes, with Col-1, OCN and OPN genes expressed at approximately three times the level of the control. These genes were closely associated with osteoblasts mineralization. Thus, PLLA-2-20 showed a higher potential for late osteogenesis, consistent with ARS staining results. In addition, the Bmp-2, Runx-2 and TGF-β signaling pathways, actively involved in and controlled bone growth, were significantly upregulated in the PLLA-2-15, with gene expression levels 2–3 times higher than those in the control and PLLA-2-0.

In conclusion, PLLA nanofiber membranes could generate electrical microenvironments upon mechanical pressure, with high piezoelectric constants providing favorable biochemical and electroactive signals for osteogenic differentiation. At the same time, low-intensity piezoelectric stimulation was detrimental to the induction of osteogenic differentiation. Therefore, PLLA-2-20 with *d*_33_ value of 14.7 pC/N and PLLA-2-10 with a piezoelectric constant of 5.6 pC/N were selected for i*n vivo* experiments.

### *In vivo* evaluation of bone regeneration capacity electrically stimulated by piezoelectric PLLA nanofiber membranes

**Figure 8.**
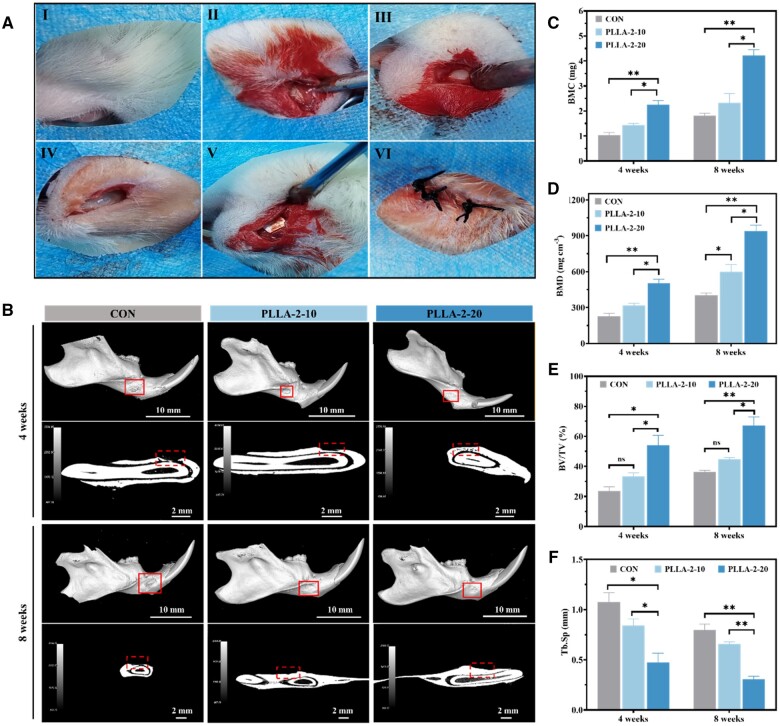
Bone regeneration electrically stimulated by PLLA piezoelectric nanofiber membranes in a rat mandibular defect. (**A**) Procedure for creating a rat mandibular defect model (3 × 1.5 mm^2^). (**B**) Micro-CT 3D reconstruction images of the defect site treated with CON, PLLA-2-10 and PLLA-2-20 at 4- and 8-week post-treatment. Quantitative analysis of (**C**) BMC, (**D**) BMD, (**E**) BV/TV and (**F**) Tb. Sp (mean ± SD, *n* = 6, *P < 0.05, **P < 0.01).

A critical-size (3 × 1.5 mm^2^) rat mandibular defect model was created by surgically creating defects (Figure 8A). Micro-CT analysis (Figure 8B) at 4- and 8-week post-surgery evaluated the bone regenerative capacity of PLLA-2-10 and PLLA-2-20. At week 4, the PLLA-2-20 with a high *d*_33_ of 14.7 pC/N, exhibited significantly formed new bone within the defect, with complete restoration at week 8, closely matching the grayscale of the surrounding original bone. In contrast, the PLLA-2-10 with lower *d*_33_ of 5.6 pC/N showed only partial new bone formation after 4 weeks, with substantial new bone formation after 8 weeks. The CON group still exhibited an open surgical cavity after 8 weeks, indicating limited new bone formation. Quantitative Micro-CT analysis confirmed these observations.

The BMC values for PLLA-2-20 were about 2-fold increase over the CON and PLLA-2-10 groups (Figure 8C). BMD in the PLLA-2-20 was as high as 477.8 g·cm^−3^ at week 4. It was increased further at week 8, leading to an increase in the grayness of the newly formed bone (Figure 8D). Bone volume fraction (BV/TV) indicated that the PLLA-2-20 group had formed around 50% new bone by week 4, which was 2.7 and 1.7 times greater than that the CON and PLLA-2-10 groups, respectively (Figure 8E). At week 8, this percentage of new bone in PLLA2-2-0 was further increased to 68.2%. Trabecular separation (Tb.Sp), a measure of the continuity of new bone, was only 0.29 mm in the PLLA-2-20 group at week 8, compared to 0.81 and 0.65 mm, respectively in the CON and PLLA-2-10 at the same time (Figure 8F).

**Figure 9.**
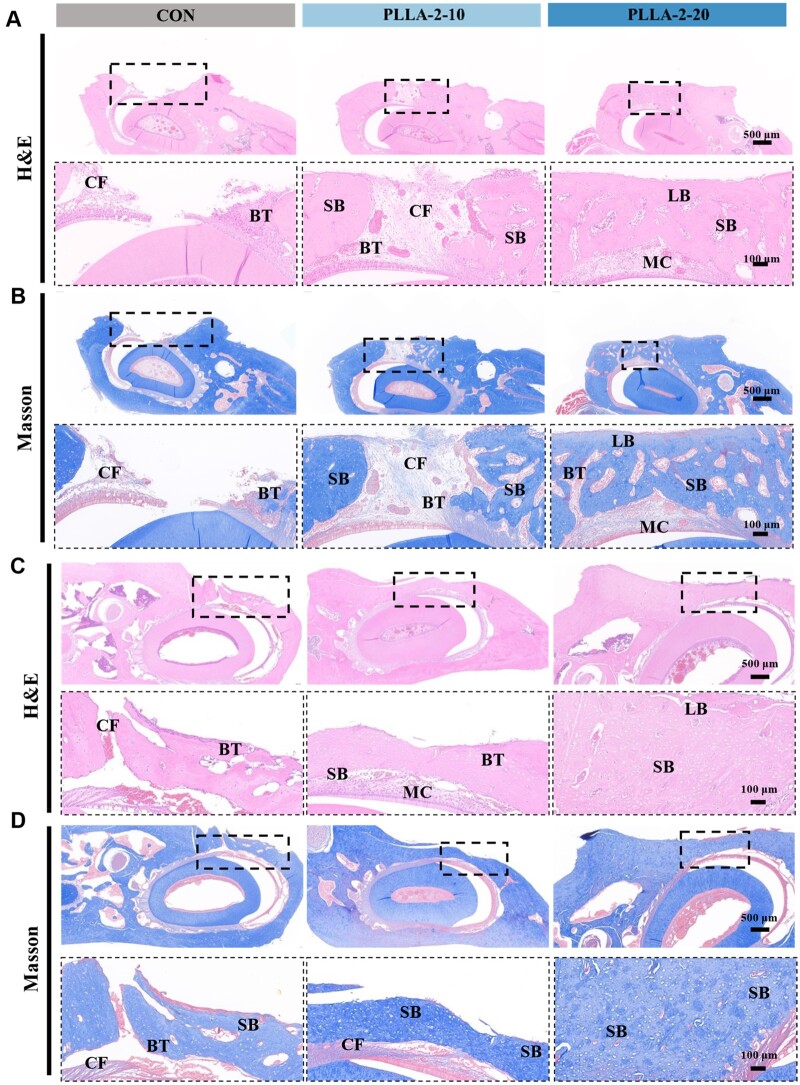
Histological assessment of the bone repair capacity of PLLA nanofiber membranes. (**A**) H&E staining showed mandibular defects in the PLLA-2-10 and PLLA-2-20 at week 4. (**B**) Masson staining characterizing the effect of mandibular repair in PLLA-2-10 and PLLA-2-20 at week 4. (**C**) H&E staining showed mandibular defects in the PLLA-2-10 and PLLA-2-20 at week 8. (**D**) Masson staining characterizing the effect of mandibular repair in PLLA-2-10 and PLLA-2-20 at week 8 (CF: collagen fibers; BT: bone trabeculae; SB: spongy bone; LB: lamellar bone; MC: marrow cavity, *n* = 6).

Histological analysis of the mandibular defect was conducted using H&E and MTC staining (Figure 9A–D). At week 4, the CON group exhibited a clearly visible surgical cavity with fibrous tissue and sparse trabecular bone structures confined to the defect’s periphery, which was consistent with the results of Micro-CT analysis. Although the PLLA-2-10 (*d*_33_ = 5.6 pC/N) demonstrated early spongy bone structure and increased density around the implant site, complete defect repair was not observed. In contrast, the PLLA-2-20 group (*d*_33_ = 14.7 pC/N) displayed a notable continuity and integrity, with the surgical cavity completely filled with a substantial volume of spongy bone. Notably, the PLLA-2-20 group also featured the presence of newborn lamellar bone and marrow cavity structures that were closely integrated with the surrounding natural bone. With the prolongation of the repair period, the ends of the defect in the CON group were bridged by trabecular and cancellous bone at week 8. In contrast, the new bone tissue in the PLLA-2-10 and PLLA-2-20 groups had completely filled the surgical cavity. Notably, the new bone tissue was denser in the PLLA-2-20 group, which had a piezoelectric constant of 14.7 pC/N, and the results of H&E and Masson staining were consistent with those of the original bone tissue. The results suggest that the self-powered piezoelectric stimulation from the PLLA-2-20 nanofiber membrane with a high piezoelectric constant significantly contributes to bone remodeling under endogenous mechanical forces.

These oriented PLLA nanofibers could guide stem cell migration and initiate the bridging process, providing a favorable platform for bone regeneration. In addition, the PLLA-2-10 and PLLA-2-20 groups, which with their distinct piezoelectric constants, successfully generated different electrical signals in response to internal mechanical stimulation, thereby affecting the bone repair process. PLLA-2-20 nanofiber membranes with the piezoelectric constant of 14.7 pC/N, were identified as providing optimal self-powered stimulation conducive to effective bone repair.

## Conclusions

Biodegradable oriented poly(lactic acid) (PLLA) nanofibrous membranes with tailored piezoelectric constants were successfully fabricated via electrostatic spinning and subsequent stabilization. By adjusting the molecular weight of PLLA as well as the concentration, volume and conductivity of the spinning solution, the piezoelectric constants of PLLA nanofibrous membranes were made adjustable and controllable from 0 to 30 pC/N. The results showed that the cell behavior was affected by the piezoelectric constant. The highest cell migration rate was observed on PLLA-2-15 membrane, which showed a 7-fold increase over the control. PLLA-2-20 membrane exhibited the optimal ability to proliferate and osteogenic differentiate the BMSCs. The PLLA-2-20 nanofiber membrane with a piezoelectric constant of 14.7 pC/N provided appropriate electrical stimulation in response to endogenous mechanical forces during the repair of a critical defect in the rat mandible, effectively promoting bone repair.

The high and tunable piezoelectric constants of the PLLA nanofiber membranes paved the way for the development of self-powered PLLA with predetermined piezoelectric properties, which could induce cell-specific behaviors.

## Supplementary Material

rbae150_Supplementary_Data
