# Supplementary material for: Tunable piezoelectric PLLA nanofiber membranes for enhanced mandibular repair with optimal self-powering stimulation
Source: Regen Biomater. 2024 Dec 26;12:rbae150. doi: 10.1093/rb/rbae150 (PMC11855284; doi:10.1093/rb/rbae150)
Supplement: rbae150_Supplementary_Data [file rbae150_supplementary_data.zip › 4b708_Supplementary material.docx]

supplementary material

**Tunable Piezoelectric PLLA Nanofiber Membranes for Enhanced Mandibular Repair with Optimal Self-powering Stimulation**

**Shuo Chen ^b^, Xinqing Wang ^b^, Dong Zhang ^b^, Zhenhua Huang ^b^, Yina Xie ^b^, Fangping Chen ^a, b*^, Changsheng Liu ^a, b*^**

*a Key Laboratory for Ultrafine Materials of Ministry of Education, School of Materials Science and Engineering, East China University of Science and Technology, Shanghai 200237, P. R. China*

*b Engineering Research Center for Biomedical Materials of Ministry of Education, East China University of Science and Technology, Shanghai 200237, P. R. China*

**Corresponding author*

*E-mail: liucs@ecust.edu.cn (C.Liu), fpchen@ecust.edu.cn (F.Chen).*

**
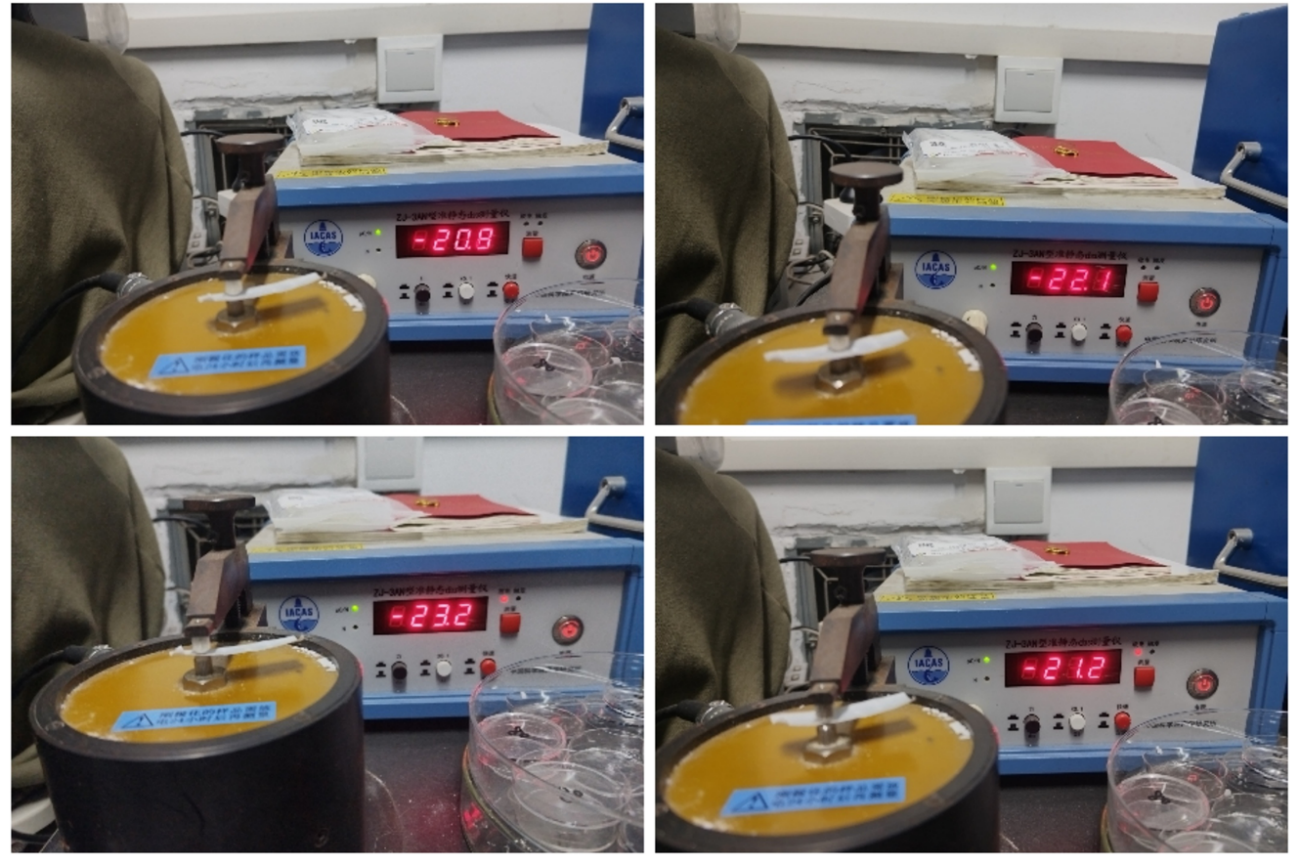
**

**Fig. S1.** Piezoelectric properties of PLLA nanofiber membranes were tested by d_33_ measurement.

**Table 1** The sequence of the genes investigated

| Name | Forward primer | Reverse primer |
| --- | --- | --- |
| ALP | TGACCTTCTCTCCTCCATCC | CTTCCTGGGAGTCTCATCCT |
| Bmp-2 | CGCCTCACAAACAACCACAG | AATGACTCGGTTGGTCTCGG |
| Col-1 | CTGCCCAGAAGAATATGTATCACC | GAAGCAAAGTTTCCTCCAAGACC |
| OCN | GAGCTGCCCTGCACTGGGTG | TGGCCCCAGACCTCTTCCCG |
| OPN | CATACAAGGCCATCCCCGTT | ACGGCTGTCCCAATCAGAAG |
| Runx-2 | TCCTGTAGATCCGAGCACCA | CTGCTGCTGTTGTTGCTGTT |
| TGF-β | ATACGCCTGAGTGGCTGTCT | TGGGACTGATCCCATTGATT |
| GADPH | CCTGCACCACCAACTGCTTA | GGCCATCCACAGTCTTCTGAG |


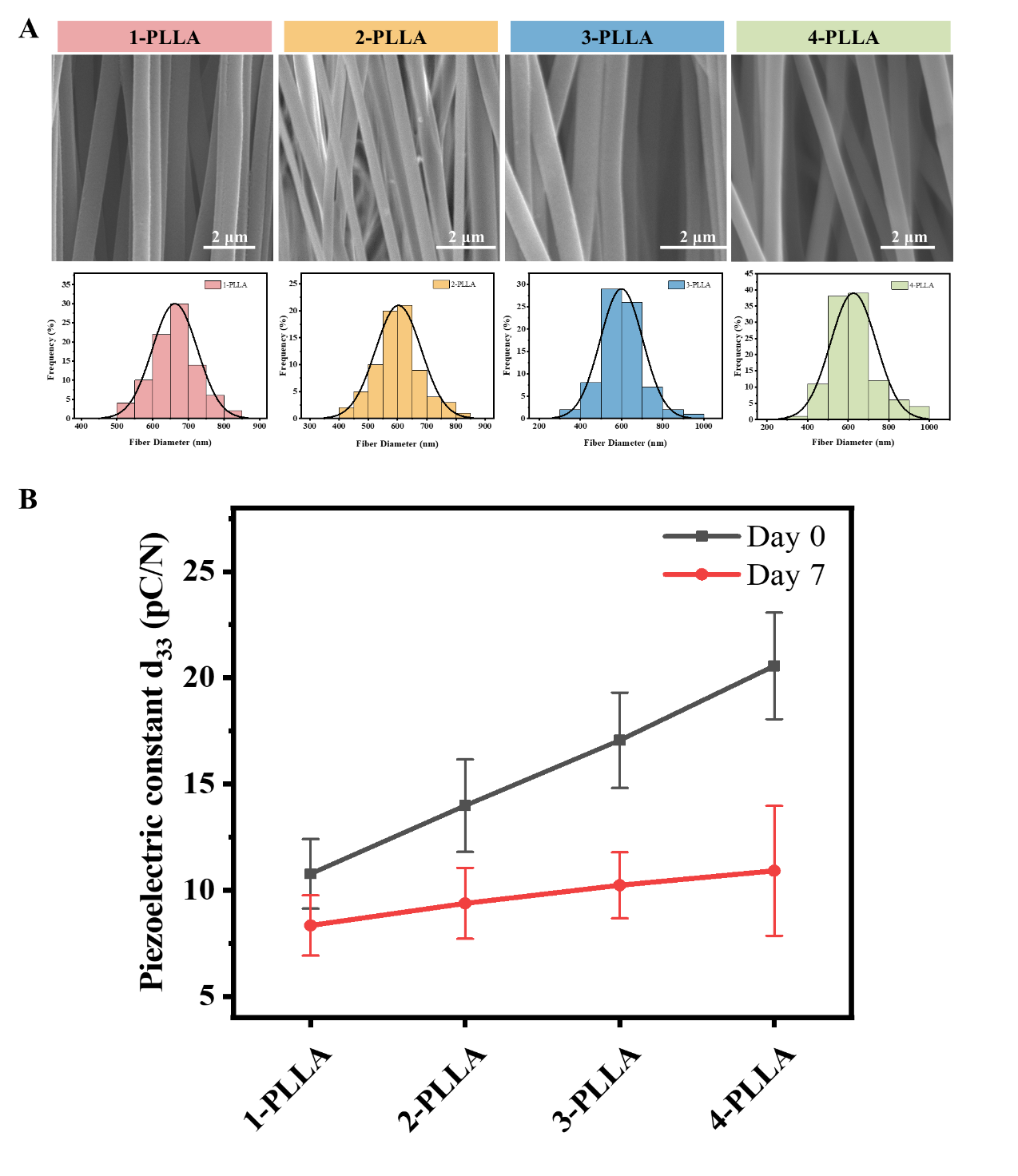


**Fig. S2.** Microscopic morphology and piezoelectric properties of PLLA nanofiber membranes. (A) Morphological observation and diameter distribution of PLLA nanofibers by modulating the melecular weight of PLLA. (B)Piezoelectric constants of PLLA nanofiber membranes controlled by modulating the melecular weight of PLLA. (mean ± s.d, n=10).

**Fig. S3.** Piezoelectric constant change curves of four PLLA nanofiber membranes with different spinning solution concentrations placed under dry conditions for 28 days.

**Table 2** Seven thicknesses of PLLA nanofiber membranes

| Name | [η] | Mw | Viscosity | Film thickness/μm |
| --- | --- | --- | --- | --- |
| PLLA-10 | 4 dl/g | 870,000 | 2 wt.% | 66.6 |
| PLLA-15 |  |  |  | 160.6 |
| PLLA-20 |  |  |  | 190.1 |
| PLLA-25 |  |  |  | 244.1 |
| PLLA-30 |  |  |  | 276.6 |
| PLLA-35 |  |  |  | 316.4 |
| PLLA-40 |  |  |  | 385.1 |

**
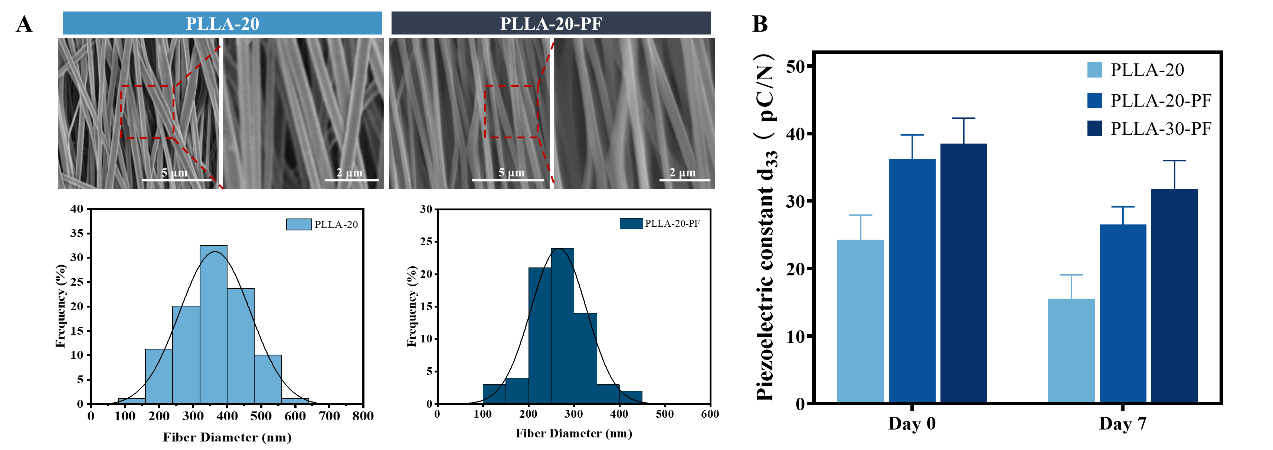
**

**Fig. S4.** Microscopic morphology and piezoelectric properties of PLLA nanofiber membranes. (A) Morphological observation and diameter distribution of PLLA nanofibers by modulating the conductive of spinning solution. (B)Piezoelectric constants of PLLA nanofiber membranes controlled by modulating the conductive of spinning solution. (mean ± s.d, n=10).

| Sample | PLLA | | Volume | PF  concentration | d_33_ |
| --- | --- | --- | --- | --- | --- |
|  | [η] | Concentration |  |  |  |
|  | dl/g | wt.% | mL | wt.% | pC/N |
| 1-PLLA | 1 | 10 | solute equal mass | - | 8.3 |
| 2-PLLA | 2 | 7 |  |  | 9.4 |
| 3-PLLA | 3 | 5 |  |  | 10.2 |
| 4-PLLA | 4 | 3 |  |  | 10.9 |
| PLLA-4 | 4 | 4 |  |  | 5.2 |
| PLLA-3 | 4 | 3 |  |  | 10.9 |
| PLLA-2 | 4 | 2 |  |  | 15.6 |
| PLLA-1 | 4 | 1 |  |  | 20.2 |
| PLLA-2-10 | 4 | 2 | 10 |  | 5.6 |
| PLLA-2-15 | 4 | 2 | 15 |  | 10.0 |
| PLLA-2-20 | 4 | 2 | 20 |  | 14.7 |
| PLLA-2-30 | 4 | 2 | 30 |  | 18.1 |
| PLLA-2-40 | 4 | 2 | 40 |  | 18.6 |
| PLLA-20-PF | 4 | 2 | 20 | 3 | 26.8 |
| PLLA-30-PF | 4 | 2 | 30 | 3 | 30.7 |

**Table 3** Experimental design of electrospun PLLA and their piezoelectric constants summary

**
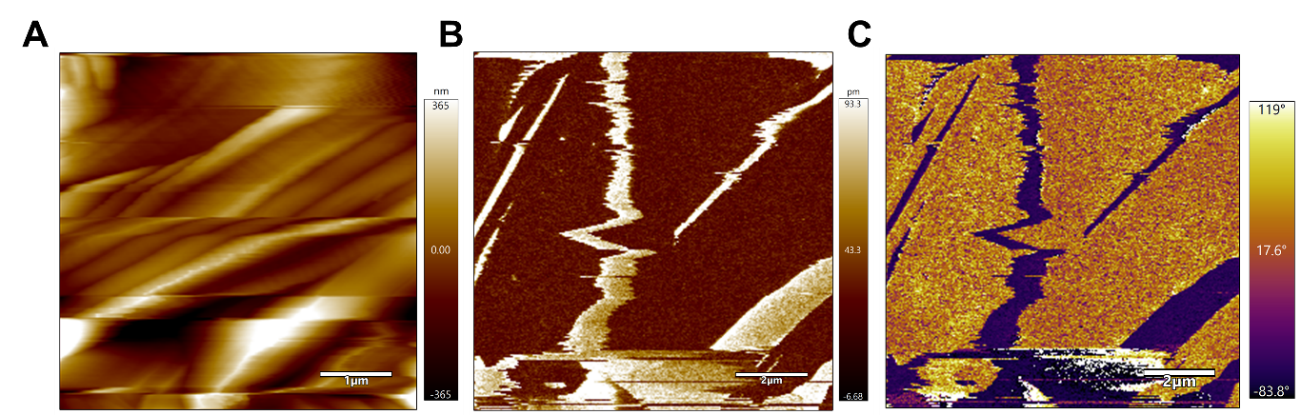
**

**Fig. S5.** Piezoelectric micrograph of PLLA-2 nanofiber membrane. (A) Morphology of PLLA-2 nanofibre. The PLLA-2 single fiber image of (B) amplitude profiles and (C) phase change profiles in response to bias.

**
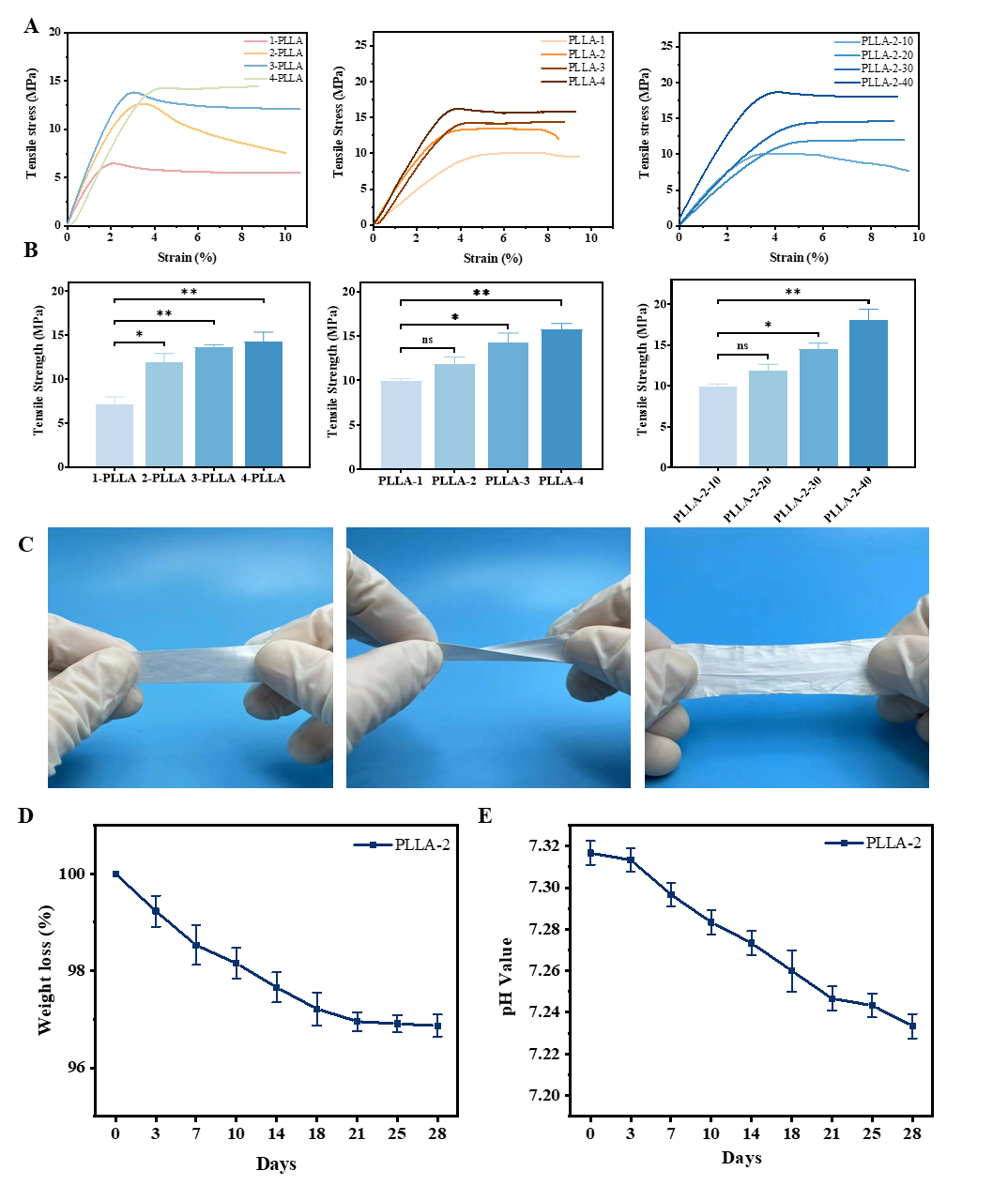
**

**Fig. S6.** Basic properties of PLLA nanofiber membranes. (A) Tensile curves of PLLA nanofibre membranes and their (B) tensile modulus statistics. (C) Digital photographs of twisted and stretched PLLA nanofibre membranes. (D) Degradation weight loss and (E) pH change curves of PLLA-2 nanofiber membranes in PBS solution. (mean ± s.d, n=3).


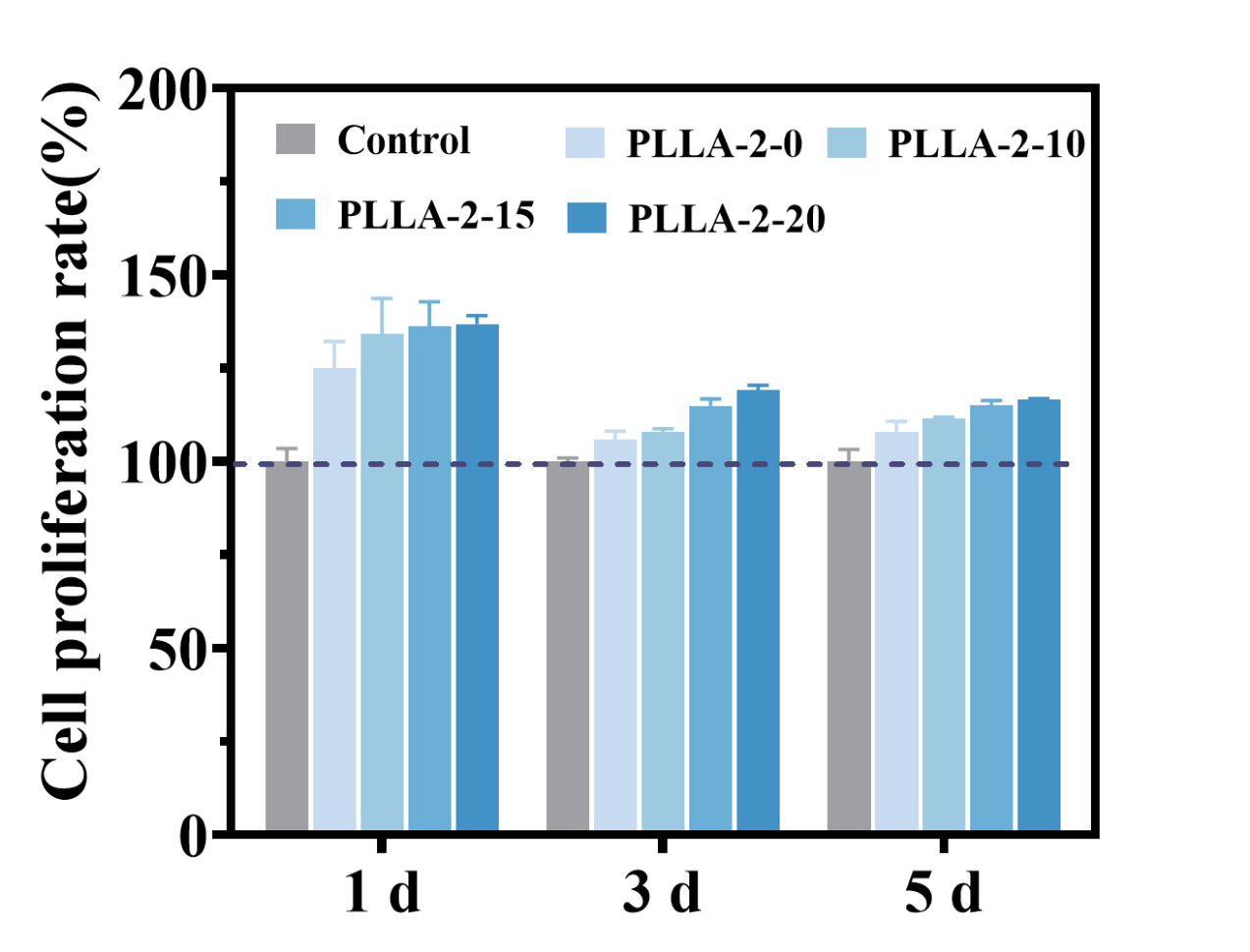


**Fig. S7.** Cell proliferation of BMSCs after 1, 3 and 5 days of culture on PLLA nanofibre membranes with different piezoelectric constants. (mean ± s.d, n=3).
